# The combined effects of napping and self-selected motivation music during warming up on cognitive and physical performance of karate athletes

**DOI:** 10.3389/fphys.2023.1214504

**Published:** 2023-07-13

**Authors:** Emna Bentouati, Mohamed Romdhani, Rihab Abid, Syrine Khemila, Sergio Garbarino, Nizar Souissi

**Affiliations:** ^1^ High Institute of Sport and Physical Education, Manouba University, Tunis, Tunisia; ^2^ Physical Activity, Sport and Health, UR18JS01, National Observatory of Sports, Tunis, Tunisia; ^3^ Interdisciplinary Laboratory in Neurosciences, Physiology and Psychology: Physical Activity, Health and Learning (LINP2), UPL, UFR STAPS (Faculty of Sport Sciences), Paris Nanterre University, Nanterre, France; ^4^ Department of Neurology, Rehabilitation, Ophthalmology, Genetics, Maternal and Child Health, University of Genoa, Genoa, Italy; ^5^ School of Occupational Medicine, Università Cattolica del Sacro Cuore, Rome, Italy

**Keywords:** alertness, cognitive functions, combat sport, daytime sleep, time to exhaustion, martial arts

## Abstract

**Introduction:** It has been established that napping or listening to motivational music during warm-up is an effective strategy to enhance cognitive and physical performances. However, which could provide better enhancement warrants further investigation. This study aimed to examine the effect of a 30-min nap opportunity (N30), a warm-up with self-selected motivational music (WUMM), and the combination of N30 with WUMM (WUMM + N30) on cognitive and physical performances in karate athletes.

**Method:** In a randomized order, 14 national-level male karate athletes performed four experimental sessions: control, N30, WUMM, and WUMM + N30. Simple (SRT) and choice (CRT) reaction times, selective attention, subjective sleepiness (ESS), mood state (POMS), countermovement jump (CMJ), and karate agility test (KAT) were evaluated before and after an all-out exhaustive task [i.e., the Karate Specific Test (KST)]. Ratings of perceived exertion (RPE) were measured immediately after the KST.

**Results:** Compared to the control, all interventions improved cognitive outcomes, mood, and sleepiness. No effects on physical performances (CMJ and KAT) were found after N30. Compared to N30, WUMM + N30 improved SRT pre- and post-exercise (pre: *p* < 0.05, d = 0.72; post: *p* < 0.001, d = 0.14), CRT (pre: *p* < 0.001, d = 0.07; post: *p* < 0.001, d = 0.10), attention (pre: *p* < 0.05, d = 0.06; post: *p* < 0.01, d = 0.06), mood (pre: *p* < 0.001, d = 2.53; post: *p* < 0.001, d = 0.23), and decreased ESS scores (pre: *p* < 0.01, d = 1.41; post: *p* < 0.05, d = 1.18). However, there was no significant difference between WUMM and N30. KST performance was not affected by the experimental conditions. However, the KST-induced performance deficit in CMJ and KAT was smaller following WUMM + N30 compared to WUMM and N30. RPE scores were lower following WUMM + N30 and WUMM.

**Conclusion:** These findings suggest that a combination of listening to self-selected motivational music during warm-up with a 30-min nap could be an effective strategy to enhance cognitive and physical performance decline caused by fatigue induced by exercise.

## Introduction

Athletes are confronted with stressful situations that disrupt their sleep and create difficulties in supporting performance and recovery, including high training loads, early morning training, exposure to altitude, competition requirements, and travel (Vitale et al., 2019). Compared to general population and sub-elite athletes, elite athletes are known to have poorer sleep quality and quantity (Romdhani et al., 2022). In this context, Souabni et al. (2022a) showed that napping can improve the recovery process, by allowing extra sleep over the course of a day. It has been reported that napping improves psychomotor (Verweij et al., 2016) and cognitive performance (Daaloul et al, 2019) and enhances short-term memory (Romdhani et al., 2021a). Previous studies have reported that napping could be beneficial to enhance performances in the 5-m shuttle run test (Abdessalem et al., 2019) and 5-m jump test (Hsouna et al., 2019) and to improve muscle strength (Tanabe et al., 2020) and endurance exercise (Blanchfield et al., 2018). Moreover, napping resulted in a better mood (Souabni et al., 2023), increased attention (Boukhris et al., 2020), and lower sleepiness (Gupta et al., 2021). Interestingly, the post-lunch (14:00–16:00) period was considered the best time for napping to reduce sleepiness (Botonis et al., 2021). In this context, it has been recommended to limit naps to 30 min to promote cognitive processes and avoid late-afternoon and evening naps that could have negative effects on night-time sleep architecture (Mesas et al., 2023). However, a 30-min nap may evoke sleep inertia, which refers to a transient decline in performance immediately after awakening (Hilditch et al., 2017). Indeed, Tanabe et al. (2020) reported that 30 min of nap included 1.4 min of a slow-wave sleep (SWS) episode compared to 13.7 min for 60 min or 16.0 min for 90 min of nap. According to Hilditch et al. (2017), shorter episodes of SWS may contribute to less pronounced sleep inertia. Interestingly, Botonis et al. (2021) highlighted that sleep inertia is more pronounced following naps of longer duration, which can be explained by sleep deprivation. Indeed, Romdhani et al. (2021a) showed that after partial sleep deprivation, repeated sprint performance was higher after a short nap than after a long nap. Additionally, several studies showed that allowing 30 min between waking up from a nap and starting the assessments could be enough to reduce sleep inertia that may have existed (Lastella et al., 2021; Daaloul et al., 2019; Romdhani et al., 2021a, b). Therefore, a shorter nap could be appropriate to avoid/minimize the risk of sleep inertia and can easily be incorporated into training/competition days, contrarily to a longer nap. Although the effects of napping on the responses to exercise after a normal sleep have been well-established, the effects of a 30-min nap, after a normal sleep, on cognitive, physical, and fatigue responses, are not well-known.

On the other hand, listening to music was a potent strategy to improve physical and cognitive performance after normal sleep (Bentouati et al., 2022). Indeed, music could present a non-invasive ergogenic aid that athletes can use during training sessions and/or competitions (Bartolomei et al., 2015). Athletes of different training statuses regularly use music to enhance performance in different exercises during their training sessions and competitions (Franco-Alvarenga et al., 2019). In addition, music preference has been shown to be an important factor in determining the ergogenic potential of music (Bentouati et al., 2022). It has been reported that self-selected motivational music improved short-term maximal exercise, rating of perceived exertion (RPE) scores, and feeling states (Khemila et al., 2021); increased heart rate and motivation (Ballmann et al., 2019); and canceled the negative impact of mental fatigue on endurance running capacity and performance (Lam et al., 2021). Interestingly, music can reduce subjective sleepiness, enhance comfort, and stimulate arousal level after a brief daytime nap (Hayashi et al., 2004). However, there is no existing study to compare the effect of napping and listening to music on subsequent cognitive and physical performances. Therefore, it is crucial to investigate the potential benefits of the combined effects of napping and listening to music and to provide practical recommendations for optimizing short naps.

Based on the aforementioned findings, napping and listening to motivational music have been shown to enhance physical and cognitive performances. However, whether napping or listening to motivational music could provide better enhancement warrants more investigation. In addition, little is known about the effect of their combination. Therefore, it seems important to investigate the effect of a 30-min nap opportunity (N30), listening to self-selected motivational music during warm-up (WUMM), and their combination on cognitive and physical performances and fatigue induced by exercise in karate athletes. It was hypothesized that 1) the combination of the nap and self-selected motivational music will result in better performance than each alone and that 2) the time to exhaustion during the Karate Specific Test (KST) will be affected by this combination.

## Materials and methods

### Participants

The sample size was *a priori* calculated using the G*Power software (Heinrich-Heine-Universität Düsseldorf, Düsseldorf, Germany) as recommended by Beck (2013) and based on the results of a study with a similar paradigm to the current one (Romdhani et al., 2021c). The choice reaction time (CRT) was selected as the main outcome. The probability of type Ⅰ (*α* ≤ 0.05) and type Ⅱ (1-β ≥ 0.95) errors was fixed at 0.05, the assumed correlation between repeated measures was 0.5, and the assumed effect size (ES) was 0.45 (Romdhani et al., 2021c). The software yielded that at least 12 participants were deemed to be sufficient to minimize the risk of incurring a type 2 statistical error with an actual power of 0.95.

Fourteen national-level male karate athletes [mean (SD) age: 19.85 ± 2.07 years; height 1.72 ± 0.06 m; body mass 65.75 ± 9.50 kg; BMI 22.05 ± 3.23 kg·m^2^] volunteered to participate and completed the protocol. After receiving a description of the protocol, potential risks and benefits of the study, participants gave their written consent to participate in this investigation. The participants were informed about their rights to leave the study at any time without any penalty. They were non-habitual nappers, non-smokers, free of drugs, and caffeine-naïve (i.e., consuming ≤80 mg of caffeine per day, according to McLellan et al. (2016). They were highly trained karateka and regularly engaged in ∼2 h per day, for at least nine sessions per week of training (including high-intensity training). All of them were of “neither type” according to the Horne and Östberg (1976) morningness/eveningness questionnaire (score between 42 and 58). Sleep diaries were collected according to the Vis–Morgen sleep questionnaire (Hohagen & Berger, 1990) (bed-time, from 22:30 h to 6:30 h ± 1:00 h), and only participants who scored ≤5 according to the Pittsburg Sleep Quality Index (Buysse et al., 1989) were included. The study protocol was conducted based on the guidelines of the Declaration of Helsinki for human experimentation (64th World Medical Association General Assembly, Fortaleza, Brazil, October 2013) and was approved by the local University Ethics Committee (CPP: N° 0113/2021).

### Experimental design

**FIGURE 1:**
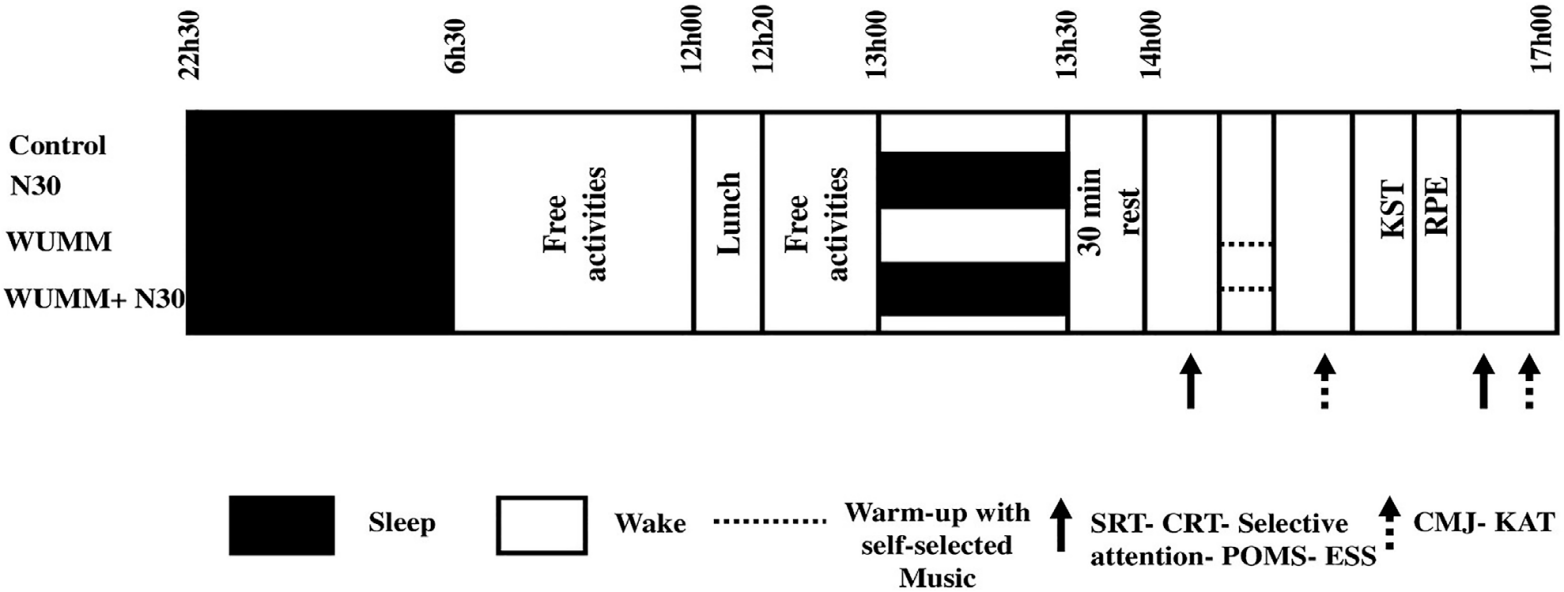
Experimental study. N30, 30 min nap; WUMM, warm-up with self-selected motivational music; WUMM + N30, a combination of warm-up with self-selected motivational music and 30 min of nap; KST, karate specific test; RPE, rating of perceived exertion; SRT, simple reaction test; CRT, choice reaction test; POMS, profile of mood state; ESS, Epworth sleepiness scale.

During the week preceding the experimental sessions, the participants were familiarized with the equipment and the experimental procedures to minimize the learning effects during the study (Figure 1). They were requested to maintain their habitual physical activity, but to avoid strenuous activity during the 24 h before the test sessions.

In a randomized and counterbalanced order, separated at least by 1 week for washout (Pincivero et al., 2001), the karate athletes participated in four test sessions: control condition, 30-min nap (N30), warm-up with self-selected motivational music without napping (WUMM), and 30-min nap before warm-up with self-selected motivational music (WUMM + N30). All experimental conditions were performed after normal sleep at night (time in bed between 22:30 h and 6:30 h). During each session, participants had the same standardized dinner at 20:00 h and went to bed at 22:30 h. It was recommended to avoid eating for 2–3 h before going to bed (Falkenberg et al., 2021). They had the same standardized breakfast at 07:00 h. They stayed awake until lunch at 12:00 h. In the time between breakfast and lunch, participants were forbidden to consume food, and only water was authorized. Participants involved in the nap conditions went to the napping room at 13:00 h for 30 min. They were controlled by two experimenters during the experiment and were awakened by an alarm placed next to the bed. Upon awakening, the participants rated their sleep quality during the nap on a 100-mm visual analog scale (Monk, 1989), ranging from 0 (no sleep at all) to 100 (deep, uninterrupted sleep), with no specific cut-off point that indicated sleep (Romdhani et al., 2021a; Souabni et al., 2022b). Participants who were unable to initiate sleep, as indicated by a score of 0 on the visual analog scale, were excluded. However, all the participants reported that they initiated sleep during the nap (N30 and WUMM + N30) and, therefore, scored >0. In the no-nap condition, participants were engaged in passive activities (e.g., watching documentaries, chatting online, or playing card games (Romdhani et al., 2021a; Romdhani et al., 2022). The post-nap test sessions started at 14:00 h, in the following order: simple (SRT) and choice (CRT) reaction time; selective attention; profile of mood state (POMS); Epworth sleepiness scale (ESS); countermovement jump (CMJ); agility test; before and after, the Karate Specific Test (KST). The ratings of perceived exertion (RPE) scores were recorded at the end of the KST.

Before the physical test sessions, participants warmed-up for 10 min, and for the music conditions, they listened to self-selected motivational music.

Laboratory conditions were set at temperature ∼19°C (±1.3°C) and humidity ∼25% (±2.3%).

### Protocol

#### Warm-up

As recommended by Tabben et al. (2014), participants warmed-up for 10 min, followed by a 5-min period of passive rest. The warm-up included self-selected intensity jogging, vertical jumping, and dynamic stretching for hip extensors, hamstrings, hip flexors, and quadriceps femoris ***.***

#### Music protocol

To mimic competitive conditions, participants listened to music only during warm-up using personal headphones. Selection criteria for music were based on the five recommendations of Karageorghis et al. (2009). In accordance with the high intensity of exercise, high tempo music (>120 to 140 beats/min) was chosen. Participants were asked to note the different genres of music they habitually listened to while exercising. According to Bentouati et al. (2022), they self-selected their most motivational songs within their top preferred genre. Participants were informed to choose a high tempo (>120 to 140 beats/min). The music was switched off at the end of the warm-up.

#### Simple reaction time (SRT)

The participants were asked to press a button as quickly as possible when the visual stimulus (green circle) appeared on the computer screen. SRT was performed using the OpenSesame software designed by Mathôt et al. (2012).

#### Choice reaction time (CRT)

This test was performed using the OpenSesame software (Mathôt et al., 2012) and consists of a colored geometric form (used as a “target”) presented to participants (Jarraya et al., 2013). There was a succession of different colored geometric forms. When the target appeared, the participant had to choose from the available possibilities to respond to the different stimuli with “A” and “P” for green and pink, respectively. Higher scores reflect poorer performance.

#### Selective attention

Three stimulus tasks were presented sequentially to the Stroop test: 1) color task, 2) words task, and 3) color–words task (Khemila et al., 2021). For the first task, the participants were asked to name just the color of each dot arranged in rows, as quickly as possible, from left to right. For the second task, the participants were asked to identify the color of ink, and not the word. For the final task, the participants were asked to identify the color of ink and not to identify or read the color or word presented. The time required to perform each task and the number of errors were recorded for each trial.

#### Profile of mood state (POMS)

To identify the mood state of the participants during each test session, the POMS questionnaire originally developed by McNair (1971), and the French version, was used in this study (Cayrou et al., 2003). This questionnaire contains 65 words and statements that describe mood, including tension, anger, fatigue, depression, confusion, and vigor. The participants rated their feelings over the time of the session on a 5-point Likert scale (i.e., ranging from 0 for “not at all” to 4 for “extremely”), and a total mood disturbance score (TMD) was calculated as follows:

TMD= (tension + anger + fatigue + depression + confusion)—vigor.

#### Subjective sleepiness

Subjective daytime sleepiness was evaluated using the Epworth sleepiness scale (ESS) as determined by Johns (1991). The participants rated their chances to fall asleep in eight different situations on a 4-point Likert scale ranging from 0 for “no chance to doze” to 3 for “high chance to doze.” If the subjective sleepiness score is higher than 6, the participant is considered sleepy.

#### Countermovement jump (CMJ)

The CMJ was performed using an optical jump system (Optojump, Microgate SRL, Italy). The participants started the test in a standing position; they quickly squatted to 90° knee flexion, followed by a jumping as high as possible while keeping their hands on their hips during the movement. Three trials were conducted, with a recovery time of 2 minutes between trials, and the better performance was selected.

#### Karate agility test (KAT)

The karate agility test is the speed of footstep and shifting directions after launching the gyaku-zuki punch (Yudhistira, 2020). This test was conducted as follows: while hearing the whistle, the participants started, from cone A, to move sideways toward B and held that cone, then they returned to cone A, then to the left toward cone C, and then to the right toward cone D.

At each sideway, the participants held the cone and performed the gyaku-zuki punch quickly. After that, the participants ran toward cone A and held cone A. Two trials with a recovery time of 6 min were recorded, and the fastest one was considered.

#### Karate specific test (KST)

The KST includes sequential sets of two attacks toward a body opponent bag. In the first attack, a two-punch combination consisted of a leading straight punch followed by a rear straight punch (kisami gyaku-zuki). In the second attack, a rear roundhouse kick (mawashi geri-chudan) was performed. The beginning of the bout of the exercise and the rest time were defined with two auditory signals. The participants performed each strike and kick with maximum possible power. The time to complete the exercise bout was 7s, and the recovery time between bouts was progressively reduced. The time to exhaustion was recorded at the end of the test when participants failed to complete the test or the power of techniques was decreased clearance (Nunan, 2006). To ensure that participants gave their maximum effort and to detect any decrease in technique quality during the KST, techniques in real-time during the test were monitored by experimenters.

#### Rating of perceived exertion scale (RPE)

At the end of the KST, the participants rated their perceived exertion using the Borg scale (Borg, 1982). The RPE consists of a 10-point Likert scale ranging from 0 for “very, very light” to 10 for “very, very heavy” efforts.

### Statistical analysis

Statistical analysis was conducted with the Statistica software (StatSoft, France), and figures were designed using GraphPad Prism 8 (GraphPad Software, San Diego, CA, United States). All values within the text and tables are reported as mean ± SD. The Shapiro–Wilk test of normality revealed that the data were normally distributed; therefore, parametric tests were performed. A two-way analysis of variance (ANOVA) (4 conditions × 2 timings [before-after the exercise]) with repeated measures on both factors was used to compare results between all test sessions (for SRT, CRT, selective attention, POMS, ESS, CMJ, and KAT). The paired sample *t*-test was used to analyze subjective sleep quality during the nap. KST and RPE scores were analyzed using one-way ANOVA. To assess the practical significance of ANOVA, the effect size was calculated as eta-squared (η^2^). The Bonferroni post-hoc test was used for pairwise comparisons, and the effect size was calculated as Cohen’s d (1992). Furthermore, the mean difference (MD) and the 95% confidence interval (95%) were provided for pairwise comparison. To evaluate the change from before to after the exercise, the delta variation (Δ%) was calculated and reported as mean ± SD. The level of statistical significance was set at *p* < 0.05.

## Results

### Sleep quality during the nap

The paired sample *t*-test showed a higher sleep quality during N30 than during WUMM + N30 (*t* = 2.13, *p* < 0.05, MD = 9.21, 95% CI = 0.09 to 18.52, mean (SD): 71.64 ± 8.36 vs*.* 60.92 ± 13.36, respectively).

### Psycho-cognitive parameters

**FIGURE 2:**
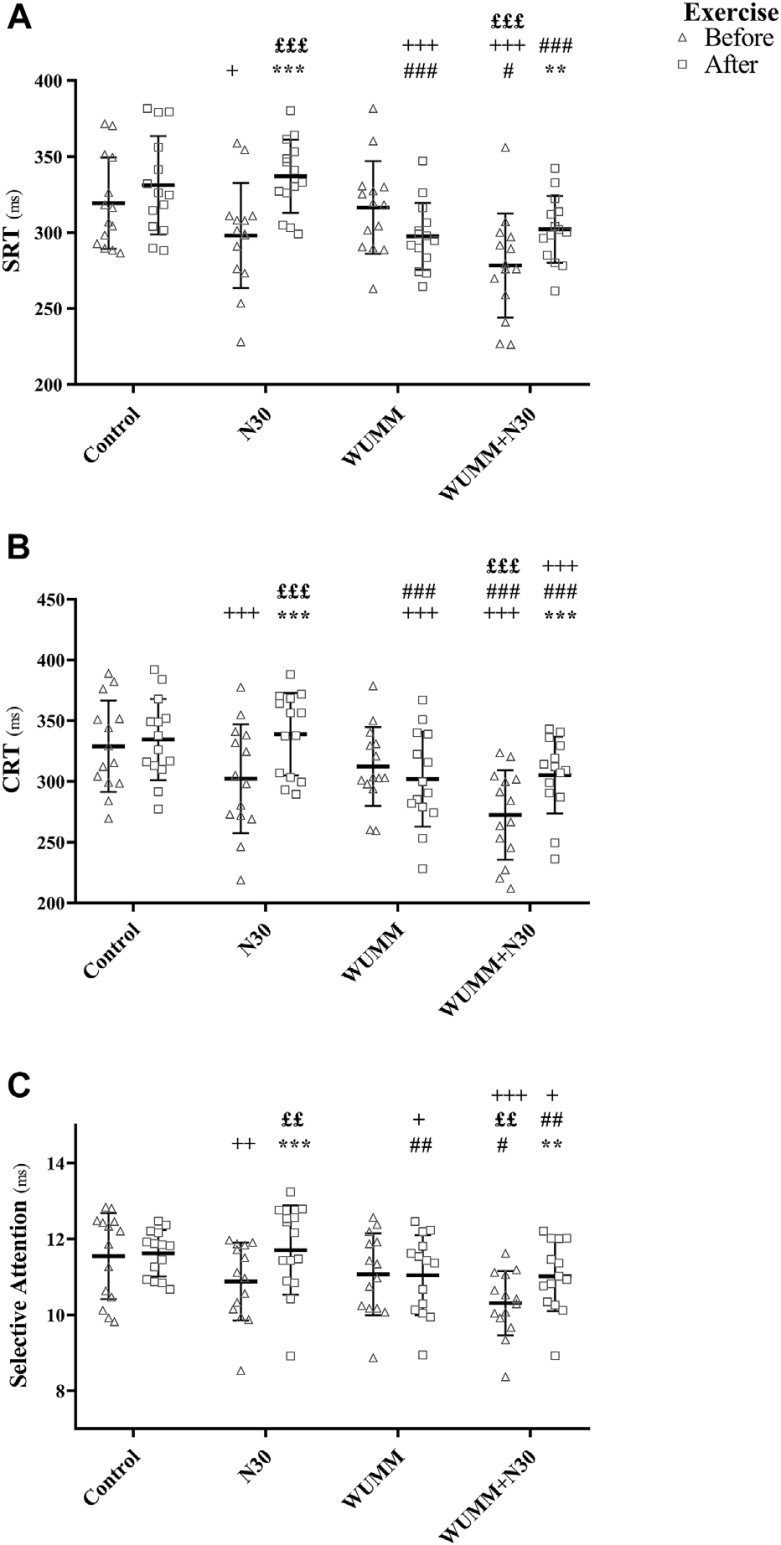
Individual values of **(A)** SRT, simple reaction time; **(B)** CRT, choice reaction time; **(C)** selective attention before and after the exercise during control, 30-min nap (N30), warm-up with self-selected motivational music (WUMM), and the combination of WUMM and N30 (WUMM + N30) sessions. ^*^, ^**^, and ^***^ indicate significant difference with before exercise at *p* < 0.05, *p* < 0.01, and *p* < 0.001, respectively; ^+^, ^++^, and ^+++^ indicate significant difference in comparison with the control at *p* < 0.05, *p* < 0.01, and *p* < 0.001, respectively; ^#^, ^##^, and ^###^ indicate significant difference in comparison with N30 at *p* < 0.05, *p* < 0.01, and *p* < 0.001, respectively; ^£^, ^££^, and ^£££^ indicate significant difference in comparison with WUMM at *p* < 0.05, *p* < 0.01, and *p* < 0.001, respectively.

**FIGURE 3:**
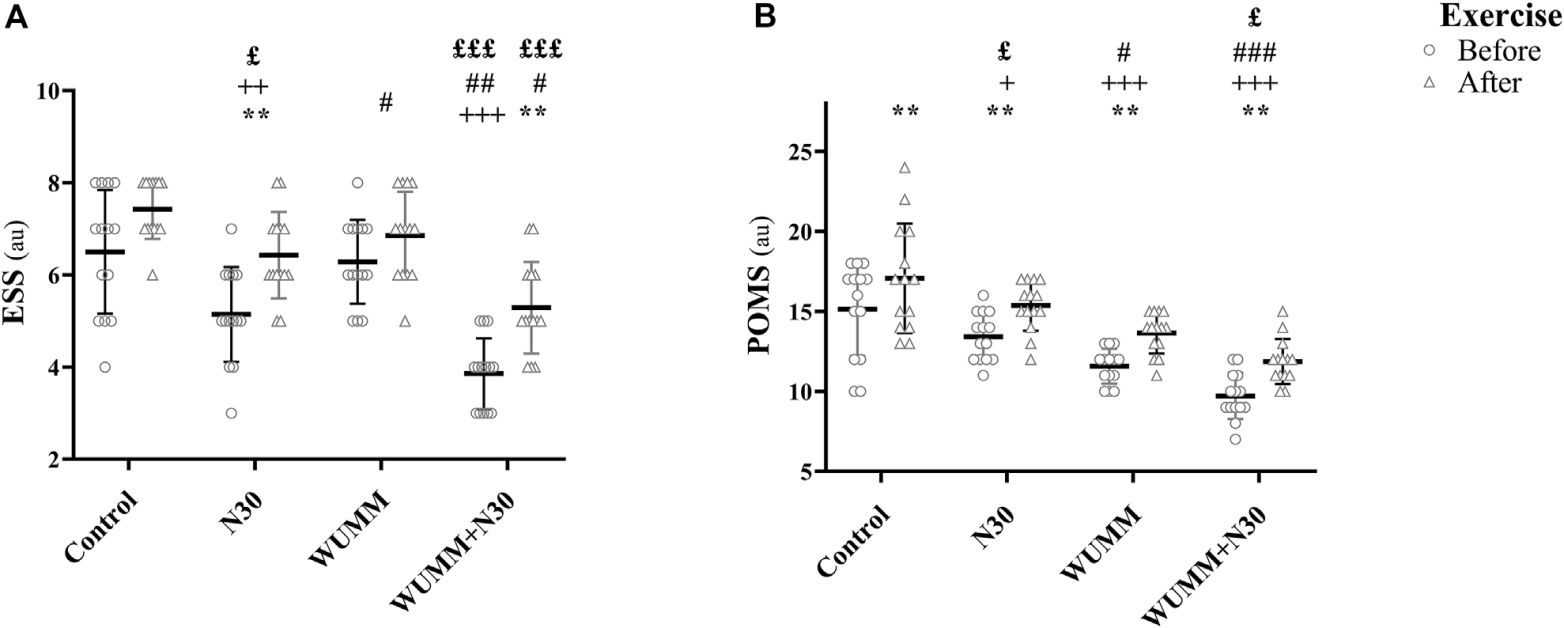
The Profile of Mood State (POMS) and Epworth Sleepiness Scale (ESS) before and after exercise during the protocol sessions. N30: 30-min nap; WUMM: warm-up with self-selected motivational music; WUMM + N30: combination of N30 with WUMM. ^*^, ^**^, ^***^: significant difference with before exercise at *p* < 0.05, *p* < 0.01, *p* < 0.001, respectively; ^+^, ^++^, ^+++^: significant difference in comparison with Control at *p* < 0.05, *p* < 0.01,*p* < 0.001, respectively; ^#^, ^##^, ^###^: significant difference in comparison with N30 at *p* < 0.05, *p* < 0.01, *p* < 0.001, respectively; ^£^, ^££^, ^£££^: significant difference in comparison with WUMM at *p* < 0.05, *p* < 0.01, *p* < 0.001, respectively.

SRT, CRT, selective attention, POMS, and ESS ANOVA and pairwise comparison results are presented in Figures 2, 3, Tables 1, 2.

**TABLE 1:** Two-way ANOVA’s output [2 (pre–post exercise) * 4 (experimental conditions)] and pairwise comparison (pre–post exercise) during each experimental condition.

	ANOVA (interaction)	Control	N30	WUMM	WUMM + N30
	F _(3,39)_; p; η^2^	d; *MD*; 95% CI	d; *MD*; 95% CI	d; *MD*; 95% CI	d; *MD*; 95% CI
SRT (ms)	18.15; <0.001; 10.09	0.51; *–11.88*; −31.22 to 7.46	1.21; *–39.02*; −58.36 to −19.68 ^***^	0.69; *18.95*; −0.39 to 38.29	0.82; *–23.78*; −43.12 to −4.43 ^**^
CRT (ms)	9.31; <0.001; 5.71	0.15; *–5.47*;-24.25 to 13.30	0.91; *–36.55*; −55.33 to −17.77 ^***^	0.28; *10.36*; −8.41 to 29.14	0.95; *–32.81*; −51.6 to −14.03 ^***^
Selective attention (ms)	6.88; <0.001; 3.18	0.08; *–0.07*; −0.62 to 0.47	0.76; *–0.82*; −1.38 to −0.27 ^***^	0.02; *0.02*; −0.53 to 0.57	0.81; *–0.71*; −1.26 to −0.15^**^
POMS (au)	0.04; NS; 0.02	0.60; *–1.92*;-3.55 to −0.3 ^**^	1.26; *–1.92*; −3.55 to −0.3 ^**^	1.75; *–2.07*; −3.69 to −0.44 ^**^	1.51; *–2.14*; −3.76 to −0.51 ^**^
ESS (au)	1.42; NS; 1.37	0.87; *–0.92*;-2.008 to 0.15	1.31; *–1.28*; −2.36 to −0.20 ^**^	0.61; *–0.57*; −1.65 to 0.50	1.61; *–1.42*; −2.50 to −0.34 ^**^

ANOVA, analyses of variance; N30, 30-min nap; WUMM, warm-up with motivational music; WUMM + N30, combination of WUMM and N30; F, Fisher’s F; p, probability; η^2^, eta-squared; d, Cohen’s effect size; MD, mean difference; 95% CI, confidence interval; NS, non-significant; SRT, simple reaction time; CRT, choice reaction time; POMS, profile of mood state; ESS, Epworth sleepiness scale.

*, **, and *** indicate significant difference with before exercise at *p* < 0.05, *p* < 0.01, and *p* < 0.001, respectively.

**TABLE 2:** Values (mean ± SD) of psycho-cognitive parameters before and after the Karate Specific Test (KST).

Parameters	Control		N30		WUMM		WUMM + N30	
	Before KST	After KST	Before KST	After KST	Before KST	After KST	Before KST	After KST
SRT (ms)	319.3 ± 30.08	331.2 ± 32.3	315.6 ± 42.9^+^	298.01 ± 34.5 ^***£££^	315.7 ± 29.8	297.5 ± 21.9^###+++^	278.3 ± 34.2^#+++£££^	302.0 ± 21.9^###**^
CRT (ms)	329.06 ± 37.5	334.5 ± 33.4	302.3 ± 44.8^+++^	338.8 ± 33.9^***£££^	312.3 ± 32.5	301.9 ± 39.2^###+++^	272.5 ± 36.9^###+++£££^	305.2 ± 31.6^###+++***^
Selective attention (ms)	11.5 ± 1.1	11.6 ± 0.6	10.8 ± 1.02^++^	11.7 ± 1.1^***££^	11.07 ± 1.07	11.04 ± 1.05^##+^	10.3 ± 0.8^#+++££^	11.1 ± 0.9^##+**^
POMS (au)	15.1 ± 2.9	17.07 ± 2.4^**^	13.4 ± 1.5^**+£^	15.3 ± 1.5^**+£^	11.6 ± 1.1^#+++**^	13.6 ± 1.3^#+++**^	9.7 ± 1.4^###+++£**^	11.8 ± 1.4^###+++£**^
ESS (au)	6.5 ± 1.3	7.4 ± 0.6	5.1 ± 1.02^++£^	6.4 ± 0.9^**^	3.8 ± 0.7^#^	5.2 ± 0.9	6.2 ± 0.9^##+++£££^	6.9 ± 0.9^#£££**^

N30, 30-min nap; WUMM, warm-up with self-selected motivational music; WUMM + N30, combination of N30 with WUMM; KST, Karate Specific Test; SRT, simple reaction time; CRT, choice reaction time; POMS, profile of mood state; ESS, Epworth sleepiness scale.

^*^, ^**^, and ^***^ indicate significant difference with before exercise at *p* < 0.05, *p* < 0.01, and *p* < 0.001, respectively,

^+^, ^++^, and ^+++^ indicate significant difference in comparison with the control at *p* < 0.05, *p* < 0.01, and *p* < 0.001, respectively,

^#^, ^##^, and ^###^ indicate significant difference in comparison with N30 at *p* < 0.05, *p* < 0.01, and *p* < 0.001, respectively,

^£^, ^££^, and ^£££^ indicate significant difference in comparison with WUMM, at *p* < 0.05, *p* < 0.01, and *p* < 0.001, respectively.

Before exercise, SRT and CRT were shorter during N30 and WUMM + N30 compared to post-exercise sessions, indicating better performances. These enhanced performances were more marked after N30 (SRT: *p* < 0.001, Δ% = 12.87 ± 11.08; CRT: *p* < 0.001, Δ% = 13.17 ± 10.63) compared to WUMM + N30 (SRT: *p* < 0.01, Δ% = 9.46 ± 10.21; CRT: *p* < 0.001, Δ% = 12.80 ± 9.85). Similarly, selective attention was greater with all the experimental conditions, except WUMM, compared to post-exercise sessions. It was better with WUMM + N30 (*p* < 0.01, Δ% = 7.63 ± 4.35) and N30 (*p* < 0.001, Δ% = 7.14 ± 7.73) compared to WUMM (*p* > 0.05, Δ% = 0.11 ± 3.74). POMS scores were lower after all conditions, and better mood states were reported after WUMM + N30 (*p* < 0.01, Δ% = 23.03 ± 11.20) compared to WUMM (*p* < 0.01, Δ% = 18.85 ± 16.02) and N30 (*p* < 0.01, Δ% = 14.56 ± 5.30). Pre-exercise ESS scores were lower after N30 and WUMM + N30 compared to post-exercise sessions, indicating reduced sleepiness. Furthermore, daytime sleepiness was lower after WUMM + N30 (*p* < 0.01, Δ% = 39.40 ± 24.32) compared to N30 (*p* < 0.01, Δ% = 29.35 ± 29.83).

### Physical performance

#### Counter movement jump (CMJ)

Performances in CMJ were significantly higher than post-exercise performances only after WUMM and WUMM + N30 conditions (Table 3). WUMM improved more CMJ performances (*p* < 0.001, Δ% = 4.11 ± 2.28) than WUMM + N30 (*p* < 0.05, Δ% = 2.87 ± 1.43).

**TABLE 3:** Values (mean ± SD) of counter movement jump (CMJ) and karate agility test (KAT) before and after the Karate Specific Test (KST).

	CMJ		KAT	
	Before KST	After KST	Before KST	After KST
Control	35.3 ± 2.8	34.9 ± 3.4	5.6 ± 0.6	5.9 ± 0.6
N30	34.8 ± 2.6^**£££**^	34.9 ± 2.2^**£**^	5.6 ± 0.5^**£££**^	5.8 ± 0.5^**££**^
WUMM	38.6 ± 2.5^**###+++£££**^	37.02 ± 2.2^***###+++£^	5.3 ± 0.5^**###+++**^	5.6 ± 0.5^*##+++^
WUMM + N30	37.0 ± 2.7^**###+++**^	36.01 ± 2.7^*#+^	5.1 ± 0.5^**###+++££**^	5.3 ± 0.4^***###+++£££^

N30, 30-min nap; WUMM, warm-up with self-selected motivational music; WUMM + N30, combination of N30 with WUMM.

^*^, ^**^, and ^***^ indicate significant difference with before exercise at *p* < 0.05, *p* < 0.01, and *p* < 0.001, respectively,

^+^, ^++^, and ^+++^ indicate significant difference in comparison with the control at *p* < 0.05, *p* < 0.01, and *p* < 0.001, respectively,

^#^, ^##^, and ^###^ indicate significant difference in comparison with N30 at *p* < 0.05, *p* < 0.01, and *p* < 0.001, respectively,

^£^, ^££^, and ^£££^ indicate significant difference in comparison with WUMM, at *p* < 0.05, *p* < 0.01, and *p* < 0.001, respectively.

#### Karate agility test (KAT)

The post-hoc test revealed that KAT performances were better before exercise than after exercise in all conditions, except N30 (Table 3). Moreover, performances were significantly higher with WUMM + N30 (*p* < 0.01, Δ% = 3.92 ± 1.45) than with N30 (*p* > 0.05, Δ% = 2.87 ± 3.86). This improvement was more marked after WUMM (*p* < 0.001, Δ% = 6.16 ± 2.22).

#### Time to exhaustion during the KST

**FIGURE 4:**
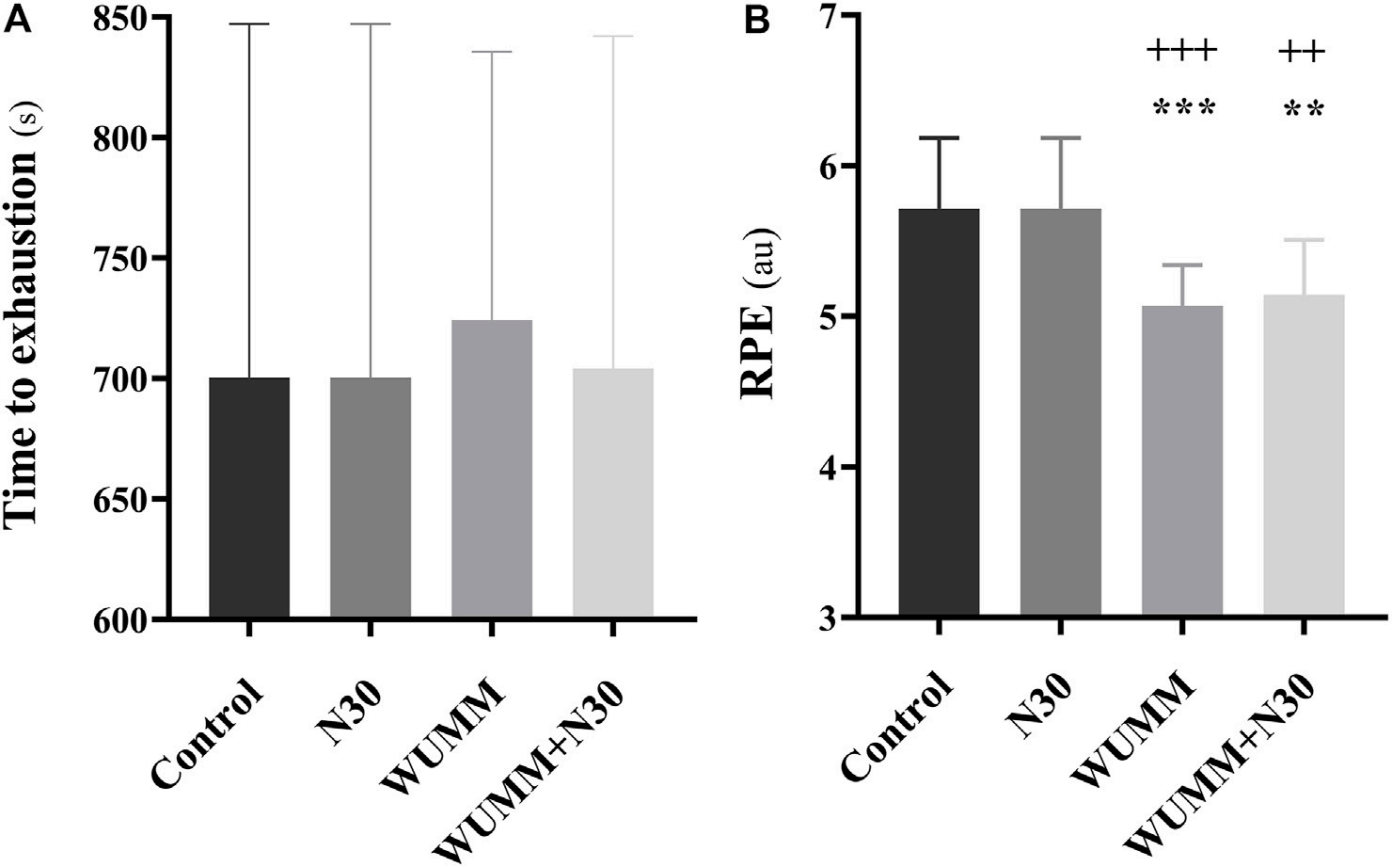
Time to exhaustion during the Karate Specific Test (KST) and Rating of perceived exertion (RPE) scores (mean ± SD) recorded after the KST. N30: 30-min nap; WUMM: warm-up with self-selected motivational music; WUMM + N30: combination of N30 with WUMM. *, **, ***: significant difference in comparison with N30 at *p* < 0.05, *p* < 0.01, *p* < 0.001, respectively; +, ++, +++: significant difference in comparison with control at *p* < 0.05, *p* < 0.01, *p* < 0.001, respectively.

There was no significant effect for conditions on the KST (F_(3,52)_ = 0.09; η^2^ = 0.005; *p* > 0.05), indicating that performance did not improve after the protocol sessions (Figure 4A; Table 4).

**TABLE 4:** Values (mean ± SD) of the Karate Specific Test (KST) and rating of perceived exertion (RPE) during each experimental condition.

	KST	RPE

Control	700.2 ± 146.8	5.7 ± 0.4
N30	700.2 ± 146.8	5.7 ± 0.4
WUMM	724.2 ± 111.3	5.07 ± 0.2^***+++^
WUMM + N30	704.1 ± 137.9	5.1 ± 0.3^**++^

N30, 30-min nap; WUMM, warm-up with self-selected motivational music; WUMM + N30, the combination of N30 with WUMM; KST, Karate Specific Test; RPE, rating of perceived exertion.

*, **, and *** indicate significant difference in comparison with N30 at *p* < 0.05, *p* < 0.01, and *p* < 0.001, respectively,

+, ++, and +++ indicate significant difference in comparison with the control at *p* < 0.05, *p* < 0.01, and *p* < 0.001, respectively.

#### Rating of perceived exertion scale (RPE)

A significant effect of conditions on RPE (F_(3,52)_ = 10.78; η^2^ = 0.38; *p* < 0.001) was observed. Compared to the control and N30, RPE scores were lower after WUMM (*p* < 0.001, d = 1.68, MD = 0.64, 95% CI = 0.22–1.05) and WUMM + N30 (*p* < 0.01, d = 1.36, MD = 0.57, 95% CI = 0.15–0.98). However, no significant difference was revealed between WUMM and WUMM + N30 and between control and N30 conditions (Figure 4A; Table 4).

## Discussion

This study is the first, to our knowledge, to evaluate the effects of a 30-min nap (N30), self-selected motivational music during warm-up (WUMM), and their combination (WUMM + N30) on cognitive, daytime sleepiness, fatigue, and physical performances after a normal sleep at night. The main findings were that the combination of WUMM + N30 improved selective attention, mood (POMS), sleepiness (ESS), and physical (CMJ, KAT) performances and Rating of Perceived Exertion (RPE). Cognitive outcomes (SRT and CRT) were more enhanced by N30. Moreover, listening to motivational music while warming-up enhanced CMJ and KAT and reduced the perceived exertion compared to napping. However, the time to exhaustion during the KST was unaffected by all the interventions.

The present results showed that the combination WUMM + N30 was more efficient on selective attention than WUMM and N30 conditions separately. In line with Khemila et al. (2021), the findings of the present study reported that music improved cognitive performances after normal sleep at night. In addition, simple and choice reaction times were faster after N30, in agreement with previous research (Romdhani et al., 2021c). Shorter reaction time after N30, compared to WUMM + N30, may be explained by the higher subjective sleep quality during N30. These improvements may be attributable to the time spent in non-rapid eye movement (NREM) sleep during napping, when the body is actively repairing and restoring itself (Venter, 2012). NREM plays a role in cellular repair, memory consolidation, motor skill recovery, and learning (Souabni et al., 2022a). Furthermore, naps may be associated with higher parasympathetic activity (Chen et al., 2020) that benefits executive functions.

Our findings indicate that mood state and sleepiness showed improvements after all the interventions. In fact, POMS and ESS scores decreased after N30 and WUMM and were more marked after WUMM + N30 in both pre- and post-exercise sessions, indicating better mood and lower sleepiness. These results are in accordance with those of Romdhani et al. (2021c), who reported similar findings after a 20-min nap. This could be explained, in part, by the fact that a 30-min nap decreases anxiety, fatigue, confusion, and depression after normal sleep (Souissi et al., 2020). Likewise, Mantua and Spencer (2017) reported that a mid-day nap reduces sleepiness and improves executive functions. It is possible that the time spent in NREM reduced homeostatic sleep pressure and sleepiness (Mantua & Spencer, 2017). Another plausible explanation is that napping reduces stress by decreasing cortisol levels (Botonis et al., 2021) and maximal heart rate during the exercise (Souabni et al., 2022b). Reportedly, music can stimulate arousal after a short daytime nap (Hayashi et al., 2004). In fact, each participant subjectively selected the music type that motivated him the most. Of interest, there was an inter-individual difference in the selected music, potentially amplifying the gain obtained by napping. Thus, the combination of WUMM and N30 can be more efficient at improving mood and reducing sleepiness.

Regarding physical performances, our results showed that N30 did not improve CMJ and KAT performances. The time to exhaustion during the KST was not affected after all the interventions. These results are in agreement with those of Daaloul et al. (2019) and can be explained by the repeated and tiring aspect of exercise. Another plausible explanation is that the short nap provides a short burst of energy, similar to earlier reports where a short nap enhanced Pmax and had no effects on Pmean and Pmin during a repeated sprint task (Romdhani et al., 2021a; 2022). Interestingly, the present study’s findings showed that all interventions, more markedly WUMM, reduced the KST-induced performance deficits on CMJ and KAT. In this context, the CMJ and KAT exercises were based on trials and the better performance was chosen, which could explain some of these improvements. Moreover, listening to music exerts an ergogenic effect to distract attention from sensations of exercise-induced fatigue (Terry & karageorghis, 2011). As a result, the combination of WUMM and N30 led to more significant enhancement in CMJ and KAT and attenuated the KST-induced fatigue.

Our results showed that RPE scores were lower after all interventions, except N30. These results are in accordance with those of Abdessalem et al. (2019), who found no effect of napping on RPE, but differ from those of Boukhris et al. (2019), who reported lower RPE scores after napping. These discrepancies might be associated with the duration of the nap, which was 25 min in the study of Abdessalem et al. (2019), 45 min in the study of Boukhris et al. (2019), and 30 min in the present study. In addition, this could be attributed to the time gap between the end of napping and the start of test sessions in the various studies. Indeed, this gap ranges from 80 to 200 min in the study of Abdessalem et al. (2019) and from 135 to 155 min in the study of Boukhris et al. (2019). However, the time gap between the nap and the test session is 30 min in the present study. Moreover, RPE scores were lower after WUMM and WUMM + N30, and this finding can be explained by the impact of preferred music in reducing RPE (Bentouati et al., 2022) and increasing motivation to exercise (Ballmann et al., 2019). It has been suggested that listening to preferred music may shift the focus away from discomfort of the exercise to the external music stimuli, resulting in lower RPE (Ballmann et al., 2021).

The study assumptions imply that a mid-day napping (30 min) combined with listening to self-selected motivational music lowered RPE and enhanced arousal, cognitive performances, and physical performance following the KST. This could be explained, in part, by the amount of time spent in NREM during the nap. On the other hand, this could be related to the impact of music preference on motivation (Meglic et al., 2021). Furthermore, the improvement in the physical performance after the KST could be associated to the reduction of RPE.

### Study strengths and limitations

The present study is the first to investigate the potential benefits of combining napping and listening to self-selected motivational music. The protocol of this study reflects real-life settings without any devices that could disrupt the sleep quality of athletes. Therefore, the study’s assumptions might be readily applicable during competition and/or training camps. On the other hand, the current study presents some limitations that have to be acknowledged. Sleep measurements during the night and the nap were recorded only subjectively using the Pittsburgh Sleep Quality Index (PSQI) and the Visual Analog Scale. However, including objective measures would provide stronger support to the study’s findings. Although discussed in the present study, sleep inertia has not been measured. Including measures of sleep inertia in future studies would provide a more comprehensive understanding of the effects of napping and music on post-awakening performance. Furthermore, we studied only the effect of listening to self-selected motivational music with one napping session (30 min). Variations in nap duration can lead to better results with the use of music (Souabni et al., 2022a). Additionally, the inclusion of only male karate athletes restricts the generalizability of the current assumptions to female athletes or other cohorts of athletes. Therefore, including female athletes in future studies might be of paramount importance. It has been reported that the performance benefits were more pronounced when the duration between awakening from a nap and subsequent evaluation exceeded 1 hour (Mesas et al., 2023). It could be possible that starting test sessions 1 hour after a nap might result in different outcomes. Finally, pre-intervention inter- and intra-individual variability may confound the post-intervention outcomes. Thus, the results of the present study should be treated with caution.

### Practical applications

It could be of importance to consider napping as a strategy to enhance cognitive outcomes. We refer to music preference when planning training sessions to stimulate confidence and motivation and to improve cognitive and physical performances. Nevertheless, coaching staff should promote nap and music strategies to maintain an adequate sleep and training/recovery patterns.

## Conclusion

Listening to self-selected motivational music has greater enhancing effects on physical performances and the rating of perceived exertion than a 30-min nap session. A combination of listening to self-selected motivational music during warm-up with a 30 min nap resulted in better selective attention, arousal, mood, and physical performance compared to the nap alone or listening to music alone. Therefore, listening to self-selected motivational music after a 30-min nap could be an effective ergogenic aid to enhance cognitive outcomes and reduce the perceived exertion in karate athletes (Faraut et al., 2011).

## Data Availability

The original contributions presented in the study are included in the article/Supplementary Material; further inquiries can be directed to the first author.
